# The incidence of deep vein thrombosis and pulmonary embolism with the elective use of external fixators

**DOI:** 10.1007/s11751-015-0219-1

**Published:** 2015-04-22

**Authors:** David J. S. Roberts, Anna Panagiotidou, Matthew Sewell, Peter Calder, David Goodier

**Affiliations:** London North West Healthcare NHS Trust, Harrow, UK; UCL Institute of Biomedical Engineering, London, UK; The Royal National Orthopaedic Hospital, Stanmore, Stanmore, UK

**Keywords:** Thrombosis, Prophylaxis, Frame, Elective, Incidence

## Abstract

Little evidence exists about the incidence of deep vein thrombosis (DVT) and pulmonary embolism (PE) with the use of external fixators. We investigated this in a cohort of 207 consecutive patients undergoing 258 elective frame applications by case note review. Case notes were obtained for 84 % of the sample population. The type of surgery, demographic data, thromboembolic risk factors and the incidence of DVT/PE were recorded. One patient experienced DVT (0.39 %) and one a PE (0.39 %). Both were of high risk and had received mechanical and chemical thromboprophylaxis during their inpatient stay. These complications were identified at least 3 months post-operatively. These findings help to more accurately counsel patients undergoing elective frame surgery on the risks of DVT/PE and also contribute to the discussion between surgeons about whether or not extended course chemical thromboprophylaxis would be of overall benefit.

## Introduction

Deep vein thrombosis (DVT) and pulmonary embolism (PE) are potential complications of lower limb orthopaedic surgery, and reported rates vary widely (DVT 0.33–0.40 %, PE 0.04–0.22 %) [1, 2, 5, 7]. Little evidence exists about the incidence of DVT and PE with the use of external fixators. Sems et al. [3] reported that 3 of 143 (2.1 %) patients screened with duplex ultrasonography following frame application for the temporary stabilisation of complex lower limb fractures were found to have a DVT.

The National Institute of Health and Clinical Excellence (NICE) provides guidance for thromboprophylaxis in orthopaedic surgery with specific recommendations for hip and knee arthroplasty and for hip fractures. Chemical thromboprophylaxis is recommended for 28–35 days for hip arthroplasty and following hip fracture surgery and 10–14 days following knee replacement. For other forms of orthopaedic surgery besides hip and knee arthroplasty and hip fracture surgery NICE recommends that individual patients should be assessed for thrombotic risk factors and chemical thromboprophylaxis given if risk factors for DVT/PE are present [4].

No published work exists regarding DVT/PE incidence with the elective use of external fixators. The aim of this work is to establish the rate of DVT/PE in those undergoing elective treatment with external fixators in our practice.

## Materials and methods

At the Royal National Orthopaedic Hospital, Stanmore, external fixators are used for elective surgery such as paediatric, adolescent and adult deformity correction and for treatment of fracture non-union. Thromboprophylaxis use is guided by the risk of thromboembolism and risk of bleeding in these cases with chemical thromboprophylaxis used only for individuals considered to be at high risk of thrombosis.

A database of individuals undergoing external fixator application by the senior authors is maintained prospectively. Information from this database and case notes were examined for consecutive patients from March 2005 to June 2011.

Occurrences of post-operative DVT or PE detected by ultrasound or CT angiogram were recorded. Risk factors for thromboembolism, type of thromboprophylaxis and time from end surgery to first dose of chemical thromboprophylaxis were also noted. Patient demographics (age, weight and height), indication for surgery, operation performed and length of operation were recorded. The length of operation was calculated as the period between the first incision of the surgeon and the time the patient left the operating theatre, both noted on the perioperative care record by theatre nursing staff. Although the time of surgeon completing the operation was not recorded, we considered our measure of length of operation time to be an acceptable estimate as the time between completion of surgery and the patient leaving operating theatre is typically five minutes or less.

Patients are counselled in clinic pre-operatively by a specialist nurse about practical and social aspects of undergoing treatment with an external fixator. The use of thromboprophylaxis in all cases was decided on individual risk factor assessment balanced with risk of bleeding. Where appropriate, a compression stocking and an intraoperative calf pump were used on the contralateral leg before, during and after surgery. Long surgical time as an isolated risk factor is not considered an indication for using chemical thromboprophylaxis by a senior author (PC). Mobilisation was commenced the morning after surgery and the majority of patients were permitted to bear weight fully as symptoms allowed.

The application of the external fixator was performed under general anaesthetic and fluoroscopic guidance without use of a tourniquet. Osteotomies, where needed, were made through small open incisions with multiple drill holes and completed in a controlled manner with an osteotome.

## Results

**Fig. 1:**
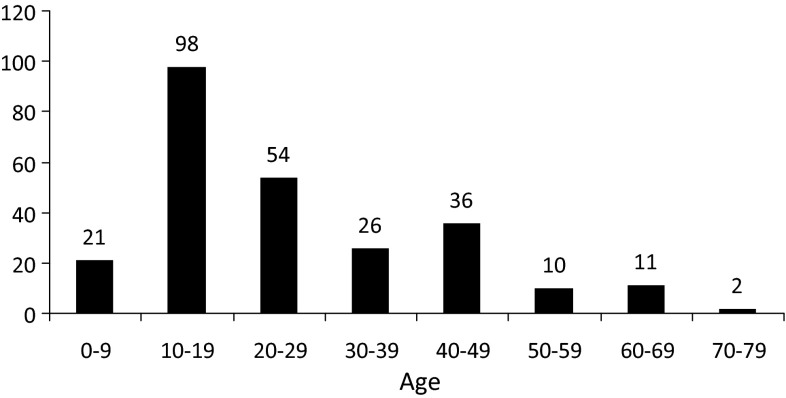
Ages of those undergoing frame application

**Fig. 2:**
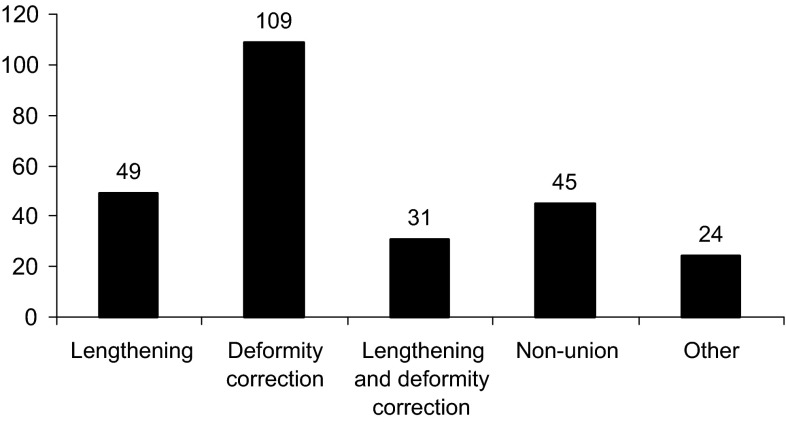
Indications for surgery

**Fig. 3:**
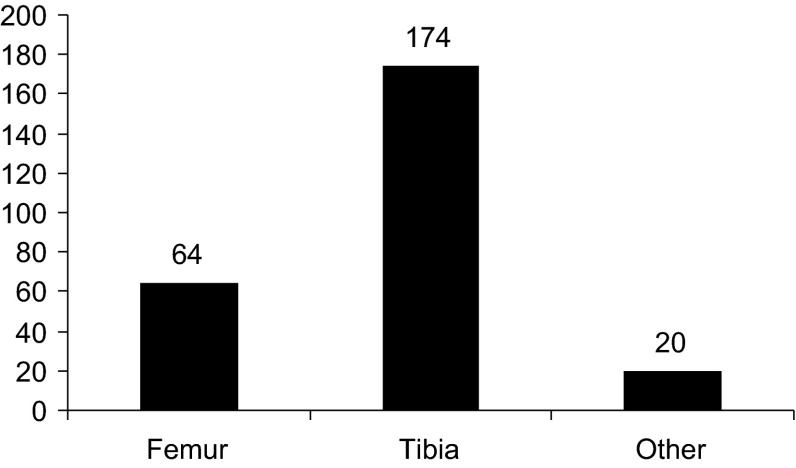
Bone to which frame applied

**Fig. 4:**
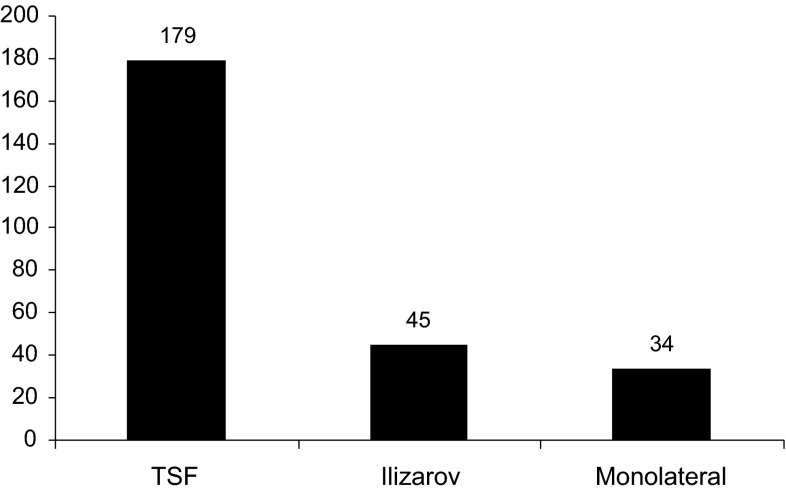
Type of external fixator

Two hundred and seven patients underwent 258 consecutive primary applications of an Ilizarov, Taylor Spatial Frame (TSF) or monolateral fixator by one of the senior authors (PC) in the study period. Data including the age of patient, indication for surgery, bone to which the frame was applied and type of fixator were available for all patients from the electronic database (Figs. 1, 2, 3, 4). Case notes were obtained for 176 individuals (84 %), representing 217 frame applications (85 %). In nine cases frames for correction of clubfoot deformity involved pins or wires in the foot with a frame bridging and immobilising the ankle. If an individual had bilateral application of external fixators under the same anaesthetic it was considered as two separate frame application events. We found no bleeding complications.

Two individuals were affected by DVT or PE. One had a DVT and one a PE, an overall isolated DVT rate of 1/258 (0.39 %) and PE rate of 1/258 (0.39 %). Considering only individuals aged 16 and older, the DVT rate was 1/183 (0.55 %) and PE 1/183 (0.55 %). In both cases, mechanical and chemical prophylaxis had been used.

### Case 1

A 43-year-old male smoker was diagnosed with non-union of a femoral shaft fracture. The fracture was sustained in July 2008 and managed with intramedullary nail fixation. For the non-union he had an unsuccessful revision nailing and subsequent drilling with bone marrow injection. In February 2009 the nail was removed and plate fixation with formal bone autograft in May 2009 resulted in bony union. Implants were removed and a unilateral external fixator was applied in January 2011 to correct shortening of the femur. He developed a DVT in April 2011 while the fixator was still in situ. He subsequently developed some symptoms of a post-phlebitic limb.

### Case 2

A 44-year-old male smoker of high BMI with a diagnosis of recurrent adamantinoma underwent excision of the lateral tibial cortex tumour and medullary curettage in April 2010. Due to a recurrence in November 2010, a segmental diaphyseal excision and application of a TSF was performed for bone transport. In the last month of a 4-month course of frame treatment he experienced a PE with symptoms of mild shortness of breath which fully resolved. Of note, this man was resident overseas and throughout the course of his frame treatment made several flights of over 3 h between the UK and his home.

**Fig. 5:**
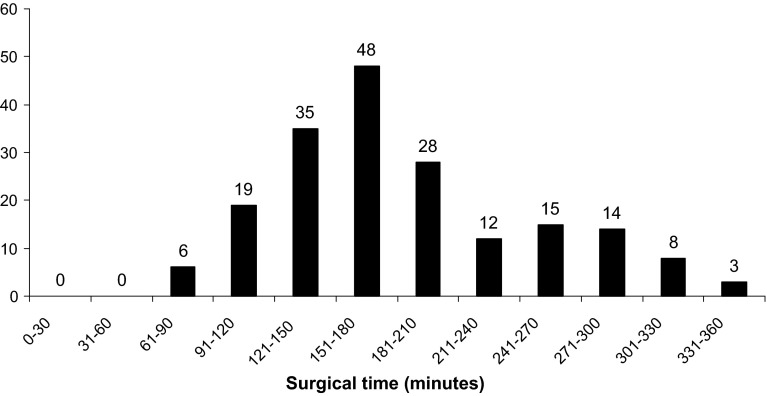
Length of procedure

Other than surgical time (Fig. 5), 47 individuals had one risk factor, six had two, and three patients had three. Risk factors are summarised in Table 1.

**Table 1:** Risk factors for DVT/PE in our population

Risk factor	Incidence
Obesity (BMI > 30)	48
Previous DVT/PE	4
Recent travel over 2 h	1
Age 60 or greater	12
Thrombophilia	1
Recent/active cancer	2

Of the procedures for which records were obtained, patients received post-operative prophylactic dose low molecular weight heparin (LMWH) for 71 procedures. Forty of those who received LMWH had identifiable risk factors on review of case notes. Of the 31 without an identified risk factor, we have postulated the potential reason for the use of chemical thromboprophylaxis which is summarised in Table 2. If the first dose of chemical prophylaxis was more than 36 h post-operatively, the use of LMWH was thought to be due to prolonged immobility. For those with insufficient data to calculate a BMI, it was surmised that this was the risk factor for which LMWH was used. One individual had polio and another who underwent simultaneous application of an ipsilateral femoral and tibial frame may have been considered to be risks of thromboembolism.

**Table 2:** Summary of risk factors in patients treated with low molecular weight heparin (LMWH)

Those with thrombotic risk factors treated with LMWH	40
Those with no thrombotic risk factors treated with LMWH	31
Postulated^a^ reason for LMWH treatment in those with no recorded risk factors	
Prolonged post-operative immobility	9
High BMI	10
Polio	1
No reason identified	11

^a^Following review of patient records

Twenty patients were found to have risk factors for thrombosis but were not treated with chemical thromboprophylaxis, summarised in Table 3. Of 12 adults with high BMI, only three had a BMI over 40; four whose only risk factor was age > 60 were aged between 62 and 66. These cases of patients with a BMI and age close to the threshold of a risk of thrombotic event may not have had their risk status noted on clinical assessment.

**Table 3:** Those individuals with risk factors for thrombosis not treated with LMWH

High BMI	12
High BMI, under 16 years old	3
Age over 60	4
High BMI and age over 60	1
Total	20

Subsequent to this study population, there have been two further cases of DVT/PE in patients treated with external fixators by the senior authors. These cases were not included in the above data, but their histories are included below (Cases 3 and 4) to help inform further discussion.

### Case 3

A 53-year-old man with knee osteoarthritis secondary to malunion of a proximal tibial fracture sustained at age 26 underwent application of a Taylor Spatial Frame for deformity correction in November 2012. The frame was removed in March 2013. Three weeks after frame removal, calf swelling prompted an investigation and a DVT was found. Following a course of anticoagulant treatment he has no residual symptoms.

### Case 4

A 30-year-old male smoker was treated with a unilateral fixator to lengthen a malunited femoral shaft fracture, which had been sustained with other injuries in a traffic collision in August 2011. He developed multiple pulmonary emboli and respiratory symptoms for 3 days following osteotomy and application of fixator in October 2012. His symptoms fully resolved. It was noted that he had reduced mobility due to his accompanying injuries.

## Discussion

The incidence of clinically significant DVT has been reported from 0.33 to 40 % with fatal PE 0.04–0.22 % in hip and knee arthroplasty [1, 2, 5, 6]. The incidence of DVT detected by screening with venogram or duplex ultrasound has been reported between 30 and 56 % [7, 8]. The work by Sems et al. [3] involved investigating all patients with duplex ultrasound following application of an external fixator as a temporary measure (2–3 weeks) in severe lower limb fractures. They found that 3 of 143 (2.1 %) patients screened with duplex ultrasonography following frame application were found to have a DVT. No bleeding complications from chemical thromboprophylaxis were experienced. This information has limited applicability to elective practice as external fixators were used in short-term stabilisation compared with the significantly longer-term use in elective surgical practice. Furthermore, trauma results in additional prothrombotic risks such as a high-energy insult to the limb, haemodynamic changes with its effect on coagulation and greater short-term immobility due to other injuries.

Efforts continue at our hospital to optimise thromboprophylaxis including optimal patient hydration, the use of mechanical thromboprophylaxis and optimal post-operative pain control to enable early mobilisation. One limitation of mechanical thromboprophylaxis in this group of patients is that the use of TEDS or intermittent compression stockings is impossible on the operated leg. Towards the end of our study period, a local Thromboprophylaxis Risk Assessment Prescribing Guide came into use and alternative tools for adolescents and children have been developed and are now used.

Limitations of this study are the retrospective collection of a significant proportion of the data, resulting in some cases of missing data. Of particular note, our postulated reasons for using chemical prophylaxis in the absence of identifiable risk factors as well as for not prescribing it for those with risk factors contribute to weakness of our data and analysis. Further confounding was from the chemical thromboprophylaxis prescription guided by individual patient factors, with some individuals given low molecular weight heparin and others not. Even so, there remains a lack of strong evidence for the routine use of chemical thromboprophylaxis in orthopaedic practice with a lack of consensus [4, 9, 10]. The aim of this work was to provide information in elective external fixator surgery to allow surgeons to inform patients of the risks of DVT and PE in this surgery and to highlight  the need for further research and discussion between clinicians about appropriate thromboprophylactic regimes.

The two patients in our study population did not become symptomatic with DVT or PE until 3 months post-operatively although it is not possible to know when the clots began, whether at the time of surgery or months after discharge from hospital. One of the two additional presented cases outside our study population (Case 3) also experienced such a delay in presentation. Therefore, if an extended course chemical thromboprophylaxis was to be used in high-risk patients, these findings suggest that it would be required for at least 3 months. Although the number of patients in our study means it is one of the largest studies of DVT and PE in elective frame surgery, these numbers are insufficient for it to provide conclusive guidance on extended course. Larger studies are required.

## Conclusion

A thromboembolic event was identified in two patients from a sample of 258 fixator applications in 207 patients. This produces an incidence of approximately 1 % of patients undergoing fixator surgery. Due to the heterogeneous manner in which prophylaxis was used, no specific conclusions can be made over the protection given by chemical or mechanical thromboprophylaxis.
